# Locoregional Considerations for Invasive Lobular Carcinoma

**DOI:** 10.1007/s12609-025-00628-8

**Published:** 2025-12-27

**Authors:** Rita A. Mukhtar, Meghan R. Flanagan

**Affiliations:** 1 Division of Surgical Oncology, University of California, San Francisco, CA USA; 2 Department of Surgery, Fred Hutchinson Cancer Center, Seattle, WA USA; 3 1825 4th St Third Floor, CA 94158 San Francisco, USA

**Keywords:** Lobular carcinoma, Invasive lobular, Lobular breast cancer, Breast surgery, Locoregional management

## Abstract

**Purpose of Review:**

This article summarizes recent literature on locoregional management of patients with invasive lobular carcinoma (ILC), including approaches to breast surgery, axillary management, and neoadjuvant therapy.

**Recent Findings:**

Breast conservation therapy is safe in ILC, but has comparatively high rates of positive margins, which can be reduced by routine use of shave margins and oncoplastic surgery. Studies demonstrating the safety of de-escalation of axillary surgery have not included enough patients with ILC to draw strong conclusions; current guidelines do not support omission of sentinel node surgery in most patients with ILC. Neoadjuvant chemotherapy may improve breast conservation and increase nodal pathologic complete response in molecularly selected patients using genomic assays.

**Summary:**

The locoregional management of patients with ILC requires special considerations based on its unique features. ILC specific studies are needed to address knowledge gaps for patients diagnosed with this common tumor type.

## Introduction

Invasive lobular carcinoma (ILC) is the second most common histologic type of breast cancer, representing 10–15% of all newly diagnosed cases [1]. Its unique features impact the locoregional management of both the breast and the axilla. Data show high rates of positive margins and completion mastectomies. Additionally, standard imaging tools have lower sensitivity in ILC, leading to understaging of disease in both the breast and axilla [2]. Large clinical trials may include patients with ILC, but histology specific findings are inconsistently reported, and studies are generally underpowered to find significant differences within ILC subsets.

In this review, we discuss the approach to surgical management of ILC in the breast, the application and limitations of axillary surgery de-escalation studies for patients with ILC, and the role of neoadjuvant therapy to potentially improve outcomes for those with ILC. While many of the principles of locoregional management of breast cancer apply to those with ILC, careful attention to the specific characteristics of lobular tumors and tailoring our treatment selection for these features could benefit patients with this common tumor type.

## Surgical Management of the Breast in Invasive Lobular Carcinoma

The unique features of invasive lobular carcinoma (ILC) require special attention when planning surgical treatment of the breast. Because ILC tumors lack the adhesion protein E-cadherin, these tumors grow in a discohesive fashion with a typical “single file” histologic growth pattern [3]. This diffuse growth pattern contributes to difficulties with detection by standard imaging such as mammography, which has a lower sensitivity for detecting ILC [2, 4]. While this can lead to delays in diagnosis, it can also lead to underestimation of true tumor size, which makes accurate surgical resection more challenging [5]. 

Multiple studies show that patients with ILC have higher rates of positive margins at breast conserving surgery (BCS) than patients with carcinoma of no special type (NST) or invasive ductal carcinoma (IDC) [6–10]. These positive margin rates range from 17 to 59%, and as expected, are higher in patients with larger tumors [11]. Consequently, patients with ILC have higher re-excision and mastectomy rates compared to those with NST tumors [12]. 

The application of margin consensus guidelines from the Society of Surgical Oncology and the American Society of Radiation Oncology to patients with ILC has resulted in a reduction in the rates of second operations [13]. Based on a meta-analysis including approximately 28,000 patients, these guidelines suggest that a margin width of “no ink on tumor” provide similar local control to wider margins [14]. While these recommendations appear to be safe in patients with ILC, it is notable that fewer than 10% of the patients included in the analysis were reported to have lobular histology; importantly, the late recurrence risk for patients with ILC suggests that longer term recurrence outcomes should be evaluated to confirm the safety of this approach [15]. 

While mastectomy may be the preferred option for some patients with ILC, multiple studies demonstrate the safety of BCS and radiotherapy, even in the setting of larger tumors [11]. Notably, data suggest that even patients who undergo mastectomy for ILC have substantial risk of positive margins given the diffuse growth pattern of this tumor type. One study found a positive margin rate of 18.7% after mastectomy for those with pathologic T3 ILC [16]. The high positive margin rates in patients with ILC across surgical procedures suggest the need for improved pre-operative imaging, surgical techniques, and consideration for neoadjuvant approaches even in the setting of planned mastectomy.

When performing BCS, both routine use of cavity shave margins and oncoplastic techniques have been shown to be associated with reduction in positive margin rates in those with ILC [17, 18]. Despite concerns about performing immediate oncoplastic surgery in the setting of higher rates of positive margins, recent data support the oncologic safety of this approach, with patients undergoing immediate oncoplastic surgery demonstrating a significantly lower positive margin rate with no difference in recurrence risk compared to standard BCS [19]. As many ILC tumors may be non-palpable, devices such as wires, magnetic or paramagnetic seeds, or radiofrequency emitting seeds may be used for tumor localization at surgery [20]. While no specific localization device has been proven to be superior for those with ILC, the use of bracketing with more than one device can facilitate larger resections and so-called “extreme oncoplasty,” allowing for greater rates of successful BCS [21–23]. 

### Role of Breast Imaging in Surgical Planning and Surveillance

Improved imaging evaluation could help improve surgical outcomes. Multiple retrospective studies have examined the utility of preoperative breast magnetic resonance imaging (MRI) in those with ILC, with inconsistent findings regarding reduction in positive margin rates or second operations [24–27]. While a meta-analysis including over 900 patients undergoing BCS with breast cancer of any type showed no difference in re-excision rates among patients who underwent pre-operative MRI, a more recent matched analysis found a significant reduction in re-excision rates in those patients with ILC who underwent breast MRI [28, 29]. 

Despite mixed data, major societies endorse the use of pre-operative breast MRI in those with ILC as it remains the most accurate imaging tool for assessing tumor extent in the breast in lobular tumors [30]. It has also been demonstrated that ILC tumors that have an imaging pattern of non-mass enhancement may be underestimated in longest dimension on breast MRI compared to those that appear mass-like, particularly after neoadjuvant therapy [31]. Additionally, breast MRI may play an important role in monitoring patients with ILC for recurrence after successful breast conserving therapy. While current recommendations from the American College of Radiology recommend supplemental MRI surveillance for breast cancer patients diagnosed before age 50 years or those with dense breasts, emerging evidence suggests that mammographic surveillance alone may be inadequate to detect local recurrence in those treated for ILC [32, 33]. Further data on the optimal surveillance strategy for these patients are needed, but tumor histology should potentially be considered when selecting surveillance imaging modalities.

While the principles of surgical management of the breast are common across all types of breast cancer, the unique features of ILC tumors require a nuanced surgical approach. Preoperative breast MRI, strategic margin management, and oncoplastic techniques can improve outcomes and reduce unnecessary mastectomies or reoperations. Additionally, appropriate selection for neoadjuvant therapy holds promise for improved rates of successful breast conservation.

### Surgical Management of the Axilla in Invasive Lobular Carcinoma

The role of axillary surgery in breast cancer is rapidly evolving. Whereas complete axillary lymph node dissection (ALND) was previously the standard of care, this has been supplanted by sentinel lymph node biopsy for clinically node negative patients [34]. When low burden of nodal disease is present in clinically node negative patients undergoing upfront surgery, radiotherapy or no further nodal-directed locoregional therapy *in lieu* of completion ALND has been demonstrated to be safe for patients with breast cancer in general. This was observed in the ACOSOG Z0011, SINODAR-ONE, AMAROS, and SENOMAC trials [35–38]. These prospective, randomized trials enrolled clinically node negative patients who were found to have 1–2 positive axillary nodes at sentinel node surgery and randomized them to either no further surgery with or without nodal radiation, or completion ALND. Omission of ALND did not result in higher rates of recurrence or diminished overall survival in these patients with largely hormone receptor positive, human epidermal growth factor-2 negative (HR+ HER2-) tumors.

For patients undergoing neoadjuvant therapy, the current standard of care remains ALND for those with residual nodal disease. However, several retrospective series question the necessity of ALND in this setting. The ongoing prospective, randomized ALLIANCE A011202 trial is testing whether nodal radiotherapy may suffice [39–41]. 

Taking de-escalation of axillary surgery one step further, recently reported randomized clinical trials such as SOUND and INSEMA have shown that in selected clinically node negative patients with low-risk disease who undergo upfront surgery, omission of axillary surgery altogether did not impact disease free survival [42, 43]. Such findings led to a recent guideline update from the American Society of Clinical Oncology supporting omission of sentinel lymph node biopsy for post-menopausal women age 50 years or older with clinical T1, grade 1–2, HR+ HER2- unifocal invasive *ductal* carcinoma with a negative pre-operative axillary ultrasound [44]. 

De-escalation of axillary surgery has been applied to patients with ILC despite higher rates of nodal involvement and decreased ability to detect nodal involvement clinically in those with ILC [45–47]. While reducing the extent of axillary surgery may reduce morbidity for patients with ILC, it remains to be seen whether the safety of this approach in those with ILC will be borne out in the long term. There are two main areas of concern arising from the unique nature of ILC tumors. First, multiple studies show that patients with ILC present with higher rates of nodal involvement than those with IDC and that this nodal involvement is harder to detect without surgical evaluation of the axilla [48, 49]. Second, as indications for certain adjuvant therapies rely upon high-risk anatomic features including nodal positivity, patients with ILC may be deemed ineligible for treatment such as CDK4/6 inhibitors, as they are less likely to have other tumor features like high grade or high Ki67 [50–52]. 

As the probability of finding additional nodal disease increases, the relative benefit of axillary surgery weighed against the risk of morbidity such as lymphedema also increases. A post-hoc analysis of the SENOMAC trial study population concluded that based on the number of patients found to have pN2-3 disease at ALND, 104 patients would have to undergo ALND to avoid one invasive disease free survival event at 5 years [53]. This ratio exposes a large number of patients to morbidity with minimal benefit for any individual patient. Interestingly, a separate post-hoc analysis of the 403 patients in the per-protocol analysis who underwent ALND on the SINODAR-ONE trial found that lobular histology was significantly associated with higher rates of pN2-3 disease [54]. With nearly a quarter of the patients with ILC having N2-3 disease in this analysis, this potentially lowers the number needed to treat to prevent one recurrence by almost half. This suggests that patients with ILC may have relatively greater benefit from more extensive axillary surgery than those with other histologic subtypes. With changes to the indications for CDK4/6 inhibitor treatment no longer requiring N2-3 disease, this concern is less relevant. However, it illustrates the need for lobular specific considerations and subset analyses so that the risks and benefits of nodal surgery can be accurately discussed with patients with ILC.

Regarding complete omission of all nodal surgery in ILC, this approach is similarly fraught by higher rates of occult nodal disease in patients with ILC [24, 55, 56]. This concern is countered by an analysis of the U.S. National Cancer Database which found that among patients age 70 or older with pT1 tumors, rates of nodal positivity were similar between those with ILC and IDC [57]. In this selected patient population, omission of sentinel node surgery for those with ILC may reduce morbidity without increasing the risk of recurrence, but limited data exist for younger patients. Of note, while ILC is known to have risk of late recurrence, de-escalation of local therapy such as radiotherapy has been shown to be associated with elevated risk of early local recurrence in those with ILC [58]. 

The premise of de-escalation of axillary surgery in breast cancer is based on the low probability of high nodal burden in certain patients combined with our ability to clinically detect such high nodal burden. While applying de-escalation to patients with ILC may be safe, it must be acknowledged that both the likelihood of high nodal burden and our ability to detect it clinically are impaired for those with this tumor type. Consequently, we need lobular specific data to allow for more tailored and evidence-based decision making.

### Neoadjuvant Therapy in Invasive Lobular Carcinoma

ILC is characterized by both histologic and genomic heterogeneity that significantly influences response to neoadjuvant therapies. The primary objectives of neoadjuvant treatment in breast cancer, whether chemotherapy or endocrine-based, are to increase the likelihood of BCS, decrease morbidity associated with ALND, and provide insight into tumor biology and long-term prognosis.

The majority of ILC cases exhibit a favorable molecular phenotype, typically comprising well-differentiated, HR + tumors with a ‘luminal A’ biology. Such tumors are generally not expected to achieve high rates of pathologic complete response (pCR) following neoadjuvant chemotherapy (NAC) [59]. Meta-analyses and retrospective studies have demonstrated lower breast and axillary pCR rates for patients with ILC compared to those with IDC [60]. However, in two studies comparing pCR rates, no statistically significant differences were observed after stratifying by hormone receptor status, suggesting that histologic subtype may not be an independent predictor of pCR [61, 62]. Indeed, several studies show higher response rates to NAC in patients with triple negative or HER2 + ILC, tumor receptor profiles that are enriched in non-classic or pleomorphic subtypes of ILC [63–67]. 

Approximately 10–20% of ILC are classified as molecular high risk and may demonstrate increased responsiveness to NAC [68–70]. Among patients with HR+ HER2- disease, high-risk genomic profiles (e.g., elevated Oncotype Recurrence Score or high-risk MammaPrint scores), progesterone receptor negativity, and poor differentiation have been associated with higher pCR rates and potential benefit from NAC [17, 64, 71]. A recent analysis from the I-SPY2 trial found that among patients with HR+ HER2- lobular histology and MammaPrint high risk tumors, nodal conversion from positive to negative occurred in 30.6% of cases. This suggests that pre-operative genomic assays may play a role in treatment selection for patients with ILC.

However, given the predominance of luminal A biology in ILC, the role of chemotherapy may be limited for many patients. In these cases, neoadjuvant endocrine therapy (NET) has demonstrated promising clinical response rates in HR+ HER2– breast cancer and may enhance breast conservation eligibility while minimizing the need for extensive axillary surgery and avoiding systemic toxicity associated with chemotherapy. Again, however, data specific to ILC within prospective and randomized NET trials remain limited, underscoring the importance of reporting outcomes in this distinct histologic subtype [72–78]. Longer duration of NET (≥ 6 months) may be associated with improved surgical outcomes among women with ILC with higher rates of breast conservation compared to shorter courses of NET; this is a valuable option for patients desiring breast conservation and reduced surgical morbidity [77, 79]. 

The ongoing Endocrine Optimization Pilot of the I-SPY2 Trial enrolls patients with clinical stage II-III breast cancer with low predicted response to chemotherapy based on genomic assay and randomizes to novel endocrine therapy-based agents with or without CDK4/6 inhibitors in the neoadjuvant setting. By assessing a variety of imaging endpoints including radiomic features on MRI and change in Ki67, decisions about adjuvant chemotherapy can potentially be based on initial response to pre-operative therapy. Such approaches may prove especially useful for patients with ILC, who often present with clinically high risk but genomically low risk disease, leading to dilemmas in treatment selection [80, 81]. 

The decision to pursue neoadjuvant therapy, whether chemotherapy or endocrine therapy, should be guided by molecular subtype given the extremely limited data on response rates specific to ILC in prospective and randomized controlled trials. Further investigation is critical to optimizing treatment strategies for this unique patient population.

## Conclusions

Ongoing efforts to refine imaging, reduce positive margins, personalize surveillance strategies, safely reduce surgical morbidity without impacting local control, and optimizing systemic therapy approaches are essential to improving care for patients with early stage ILC. Much of the available data on locoregional management for patients with ILC comes from retrospective or single institution series, highlighting the unmet need for lobular specific studies.

**Fig. 1:**
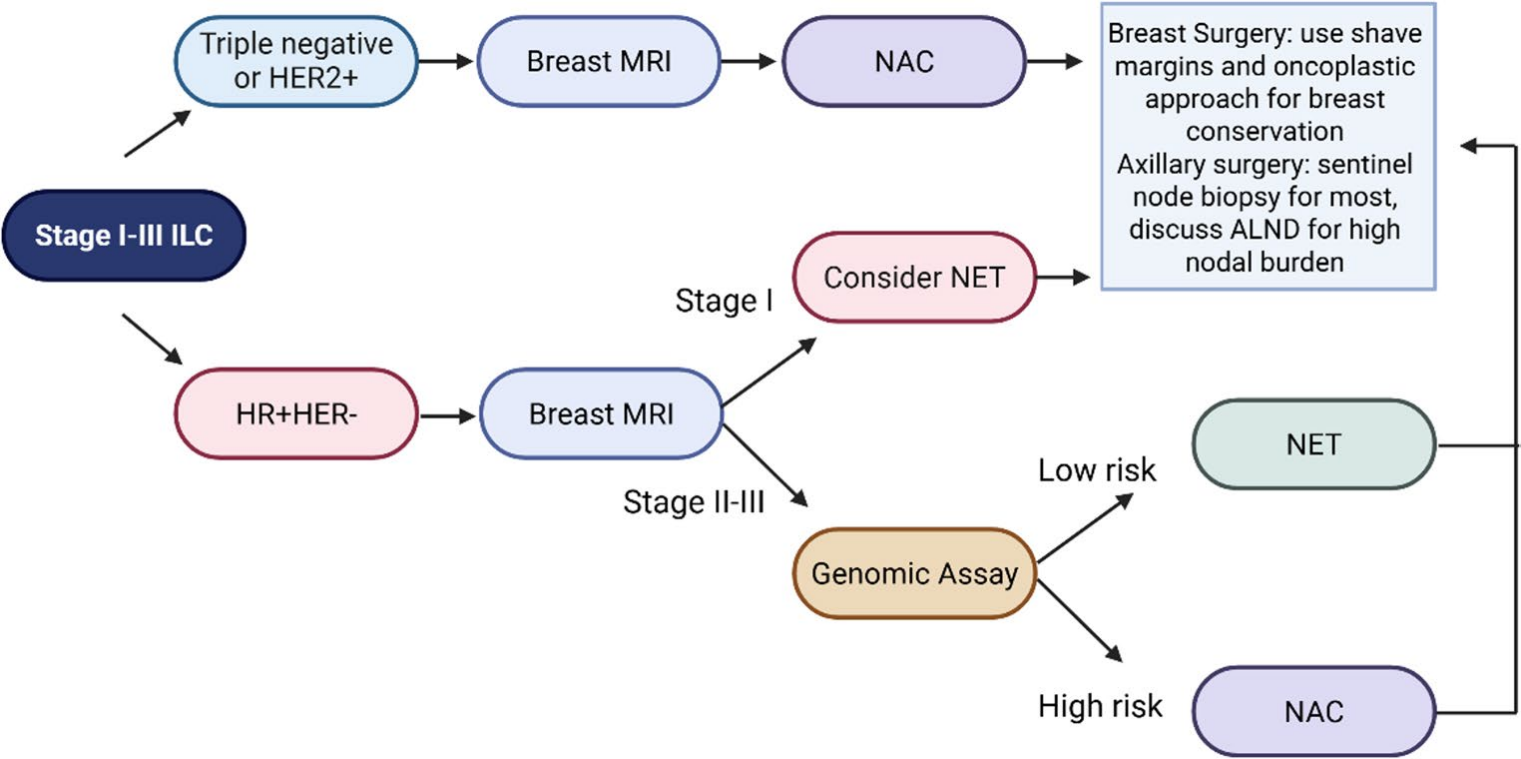
Legend: Schematic showing approach to locoregional management in patients with stage I-III ILC. For uncommon triple negative or HER2 + ILC, consider NAC but small T1 tumors may proceed to primary surgery. In general, breast MRI should be omitted if contraindication present such as chronic kidney disease or patient cannot tolerate. Tumor appearance on breast MRI (mass versus non-mass enhancement) should be considered when interpreting findings as it may impact accuracy for surgical planning. Figure created in https://BioRender.com

Given the limitations of available data, we suggest the following approach for locoregional management (Fig. 1). Most patients with early stage ILC should undergo pre-operative evaluation with breast MRI. For stage II or III tumors, pre-operative genomic assays can identify patients with molecularly high-risk disease for whom neoadjuvant chemotherapy may be considered. For those with molecularly low risk disease, long courses of neoadjuvant endocrine therapy of at least 6 months may improve successful breast conservation. This can also be improved with the routine use of shave margins and oncoplastic surgery. The pros and cons of omission of sentinel node surgery should be discussed in carefully selected patients aged 70 years or older, along with the limitations of axillary imaging in those with ILC and potential implications for adjuvant therapy. Finally, further study on surveillance imaging such as supplemental MRI is needed to help identify patients with recurrence after successful breast conserving therapy.

## Key References

- Van Baelen K, Geukens T, Maetens M, et al. Current and future diagnostic and treatment strategies for patients with invasive lobular breast cancer. *Ann Oncol Off J Eur Soc Med Oncol*. 2022;33(8):769-785. 10.1016/j.annonc.2022.05.006.
  - ○ A recent comprehensive review of treatment approaches for lobular breast cancer.
- Falade I, Switalla K, Quirarte A, et al. Oncologic Safety of Immediate Oncoplastic Surgery Compared with Standard Breast-Conserving Surgery for Patients with Invasive Lobular Carcinoma. *Ann Surg Oncol*. 2024;31(11):7409-7417. 10.1245/s10434-024-15326-5.
  - ○ These data support the use of immediate oncoplastic surgery in those with ILC, despite concerns about the safety of this approach.
- Clelland EN, Quirarte A, Rothschild HT, et al. Surveillance Strategies After Primary Treatment for Patients with Invasive Lobular Carcinoma of the Breast: Method of Local Recurrence Detection After Breast-Conserving Surgery. *Ann Surg Oncol*. 2024;31(11):7315-7322. 10.1245/s10434-024-15710-1.
  - ○ The only currently available data evaluating method of detection of recurrence in those with lobular breast cancer.

## Data Availability

No datasets were generated or analysed during the current study.
